# Complete genome sequences of bacteria isolated from cockroaches collected in a Bangladeshi hospital

**DOI:** 10.1128/MRA.00356-23

**Published:** 2023-08-22

**Authors:** Sean D. Workman, Saiful Islam, Shamima Akter, Tamanna Nasrin, Fazlul Haque, Christopher K. Yost, Moni Krishno Mohanta

**Affiliations:** 1 Institute for Microbial Systems and Society, Faculty of Science, University of Regina, Regina, Saskatchewan, Canada; 2 Department of Biology, Faculty of Science, University of Regina, Regina, Saskatchewan, Canada; 3 Genetics and Molecular Biology Laboratory, Department of Zoology, University of Rajshahi, Rajshahi, Bangladesh; 4 Microbiology Department, Faculty of Science, Chulalongkorn University, Bangkok, Thailand; Indiana University, Bloomington, Bloomington, Indiana, USA

**Keywords:** Nanopore, genome, cockroach, nosocomial, multidrug, resistant, plasmid

## Abstract

We report the complete genome sequences of four bacterial strains that were isolated from *Blattella germanica* (German cockroaches) that were found in three wards of the Rajshahi Medical College Hospital. Multiple antibiotic resistance genes were identified in each genome, with one genome containing multiple plasmid-encoded resistance genes.

## ANNOUNCEMENT

Nosocomial infections are responsible for significant morbidity and mortality and account for a substantial proportion of adverse events in hospitalized patients (1). Nosocomial infections can also have an impact at the community level as they are often linked to multidrug-resistant (MDR) bacteria (2). Cockroaches have been shown to act as reservoirs for MDR bacteria and have long been suspected to act as vectors for nosocomial pathogens (3, 4). We report the complete genome sequences of four bacterial strains harboring multiple antibiotic resistance genes isolated from cockroaches that were collected from three wards in the Rajshahi Medical College Hospital in Rajshahi, Bangladesh.

Samples of the bacteria from the external and internal surfaces of the cockroach were isolated as previously described (3). Samples were spread plated on eosin-methylene blue agar, MacConkey agar, and xylose lysine deoxycholate agar and were incubated at 37°C for 24 h to aid in the taxonomic classification of isolated bacteria. Individual colonies were subcultured on nutrient agar to obtain pure cultures. The pure cultures were grown in nutrient broth at 37°C for 24 h before cells were collected for DNA extraction. Genomic DNA from the pure cultures was isolated using the Wizard Genomic DNA Purification Kit (Promega), and whole-genome sequencing was performed to assist with final taxonomic classification and to identify antibiotic resistance genes.

Long-read sequencing libraries were prepared using the Oxford Nanopore Technologies (ONTs) Rapid Barcoding Kit (SQK-RBK004), which has been shown to recover plasmid sequences more reliably (5). Sequencing was done using ONT’s MinION sequencing platform with an R9.4.1 flow cell (FLO-MIN106D). Basecalling, demultiplexing, and adapter trimming were performed with ONT’s Guppy (6.4.2) using the Super Accuracy model. Reads were processed with Porechop (0.3.2pre) to remove any remaining adapter sequences, and the resulting reads were quality filtered with Filtlong (0.2.1) to retain the top 95% of reads based on nucleotide quality score while discarding any reads shorter than 1,000 bp. Genome assembly was carried out using Flye (2.9) using the –nano-hq and -i 3 flags to indicate the reads were generated using the Super Accuracy model and to incorporate three iterations of genome polishing (6). Output from Flye denoted circular sequences for all replicons from all genomes. The assembled genomes were polished using ONT’s Medaka (1.5.0). Genome annotation was carried out using NCBI’s PGAP pipeline (7). Antibiotic and environmental stress resistance genes were identified using NCBI’s AMRFinderPlus tool (3.11.4) (8). Taxonomic assignment was carried out using the Type (Strain) Genome Server (9), which employs a combination phylogenetic tree inference and digital DNA:DNA hybridization. Plasmid typing was carried out using PlasmidFinder (2.0.1) with default settings (10). Detailed sequencing and assembly statistics can be found in Table 1.

**TABLE 1:** Strain sequencing and assembly statistics

GenBank assembly accession no.	Species	Genome size (bp)	GC content (%)	Number of replicons	Predicted no. of CDSs	AMR genes	SRA accession no.	Nanopore barcode	Input DNA(ng)	No. of reads	Read N50(bp)	Mean coverage
GCA_029873445.1	*Serratia marcescens*	5,020,614	59.9	1	4,649	6	SRR24314885	1	400	264,244	3,689	161
GCA_029873425.1	*Enterobacter hormaechei*	5,021,643	55.3	6	4,801	18	SRR24314884	2	400	213,249	3,318	118
GCA_029873405.1	*Enterobacter cloacae*	5,177,606	54.5	3	4,845	3	SRR24314883	3	400	141,885	3,576	83
GCA_029873385.1	*Serratia marcescens*	5,220,563	59.6	1	4,851	6	SRR24314882	4	400	104,263	3,292	56

Two of the four sequenced genomes were identified as *Serratia marcescens*, one genome was identified as *Enterobacter cloacae,* and one genome was identified as *Enterobacter hormaechei*. All four genomes contained multiple antibiotic resistance genes; however, the *E. hormaechei* genome was of note because of its 18 unique resistance genes, nine of which are encoded on a single IncFIB-type plasmid with genes also coding for conjugation capabilities.

## Data Availability

Assembled genome sequence files are available under BioProject number PRJNA956928 with GenBank accession numbers GCA_029873445.1, GCA_029873425.1, GCA_029873405.1, and GCA_029873385.1. Raw sequence read files have been deposited in the NCBI Sequence Read Archive under accession numbers SRR24314885, SRR24314884, SRR24314883, and SRR24314882.
